# Trials for reducing the dangerous effect on poultry fed on aflatoxin contaminated ration using nano curcumin

**DOI:** 10.1186/s12917-025-04519-z

**Published:** 2025-02-17

**Authors:** Marwa Abo Bakr Hamouda, Adel Hassan Saad, Amr Abd El-khalik Abd El- hamid El-Saadany, Tamer Mohammed Hafez Ahmed El Sharawey, Walied Abdo, Eman Mahmoud El-diasty, Sabreen Ezzat Fadl, Ehab Yahya Abdelhiee

**Affiliations:** 1 Mycology Department, Animal Health Research Institute (AHRI), Agriculture Research Center (ARC), P.O. Box 12618, Giza, Egypt; 2 Nutrition and Clinical Nutrition Department, Faculty of Veterinary Medicine, Matrouh University, Matrouh, 51744 Egypt; 3 Mycology Department, Animal Health Research Institute (AHRI) Tanta Branch, Agriculture Research Center (ARC), P.O. Box 12618, Giza, Egypt; 4 Food Hygiene Department, Animal Health Research Institute (AHRI) Tanta Branch, Agriculture Research Center (ARC), P.O. Box 12618, Giza, Egypt; 5 Pathology Department, Faculty of Veterinary Medicine, Kafrelsheikh University, Kafr El- Shaikh, 33516 Egypt; 6 Biochemistry Dept, Faculty of Veterinary Medicine, Matrouh University, Matrouh, Egypt; 7 Forensic Medicine and Toxicology Department, Faculty of Veterinary Medicine, Matrouh University, Matrouh, 51744 Egypt

**Keywords:** Aflatoxin, Curcumin, Nano curcumin, Broilers, Residue, Mould's prevalence

## Abstract

**Background:**

Aflatoxin has a negative impact on the health of both humans and animals. One of the reasons for financial losses in the chicken sector is aflatoxicosis. In chickens, aflatoxicosis results in lowered growth rates and egg production, increased mortality, and diseases susceptibility. The current investigation sought to determine the mould’s prevalence at the Giza and Assiut Governorates. Then, the isolated toxigenic strain was used to obtain aflatoxin B1, which used to evaluate the dietary influence of curcumin and nano curcumin on growth performance, carcass traits, biochemical, aflatoxin residue, and pathological lesion of liver, spleen, and intestine in Cobb broiler chickens. 120 hatched chicks were divided into 4 group. The groups were control fed basal diet without additives, Afl group fed diet contaminated with aflatoxin, Afl + Cu group fed diet contaminated with aflatoxin and curcumin as a feed additive (7 g curcumin/kg diet), and Afl + Nano-Cu group feed diet contaminated with aflatoxin and nano curcumin as a feed additive (400 mg nano curcumin/kg diet).

**Results:**

The results indicated that curcumin better than nano curcumin in ameliorating the deleterious effects of aflatoxin that appeared in improving the body weight gain, liver function, and pathological condition of liver, spleen, and intestine than nano curcumin fed group.

**Conclusion:**

The current study offers an experimental scientific foundation for the use of curcumin as a medicinal medication or supplement in animal husbandry practices.

## Background

One of the reasons for financial losses in the chicken sector is aflatoxicosis (Tedesco et al. [64]). In chickens, aflatoxicosis results in lowered growth rates and egg production, increased mortality and diseases susceptibility, listlessness, fatty livers with poor pigmentation, anorexia, and negative feed conversions (Gholami-Ahangaran & Zia Jahromi., [19]). Ingestion of tainted food and feed is the main way that aflatoxicosis is spread. The primary biological impacts of aflatoxin are carcinogenicity, genetic modification, and teratogenicity (Mishra and Chitrangada [41]).

The golden turmeric pigments known as curcuminoids, have a wide range of physiological effects, including anti-oxidative, anti-carcinogenic, anti-inflammatory, anti-hepatotoxic, anti-microbial, antacid, radioprotective, and hypocholesterolemic properties (Gernat et al. [17]). It is regarded as *Curcuma longa’s* primary active ingredient (Flora et al. [16]). Curcumin has been shown in earlier research to shield broilers and rats against AFB1’s hepatotoxic and carcinogenic effects (Ashry et al. [6]; Poapolathep et al. [50]; respectively). But curcumin has several problems (Hewlings and Kalman [27]). One of the main issues with curcumin has been its low oral bioavailability, which is caused by low absorption, rapid metabolism, and rapid systemic excretion from the body (Rahmani et al. [51]). As a result, using curcumin nanoparticles increases its solubility in water and enhances its bioavailability in the body (Heidary et al. [25]). Moreover, absorption rate of nano curcumin is high, about 10–14-fold free curcumin (Setthacheewakul et al. [60]).

This topic has been studied a lot, but in this study, we increased the dose of aflatoxin to determine the extent of curcumin and curcumin nanoparticle’s ability to reduce aflatoxin toxicity in poultry. In addition, the isolation of the mould from the diet of poultry farms. Thus, the purpose of this study was to screen isolated mould from the diet of poultry farms for the production of aflatoxins. Additionally, to examine the effects of supplemental diets containing curcumin and nano curcumin with a high dose of aflatoxin (1 mg of AFB1/kg diet instead of 0.25 mg/kg diet) on the following aspects of broiler chicks’ diets: clinical signs, growth performance, carcass traits, serum biochemistry, aflatoxin residue, and tissue histopathology.

## Methods

### Collection of poultry ration and mould isolation

100 samples from poultry diet were collected from different poultry farms at Giza and Assiut Governorates. The collected samples were transported to Animal Health Research Institute, Dokki, Giza, Egypt in ice box. Serial dilution was done for each ration sample and cultured on Rose Bengal media. After cultivation, the cultivated samples were incubated at 25–27 ^O^C for 5:7 days to determine total number of mould.

An examination and classification of the isolated mould was carried out on the classification-specific cultures, such as Czapex yeast agar. Then, lacto phenol cotton blue dye was used for microscopic examination. The toxigenic *Aspergillus flavus (A. flavus)* was determined using Yeast sucrose broth for culture (for 21 days) to promote mycotoxin formation. The isolated strains were screened for the production of aflatoxins (AFs) using Thin Layer Chromatography (TLC) (Shotwell et al. [61]).

### Aflatoxin production and preparation of the poultry diet

After cultured, the various AFs was qualitatively confirmed by the appearance of blue fluorescence on the plate and comparison of the spot’s Retention Factor (RF) value versus the RF of a known standard. By using a TLC and physical examination, it has been determined that commercially crushed yellow corn was completely devoid of any mycotoxin or fungus contamination. Following that, the examined corn was put into the conical flasks and autoclaved for 15 min on three different days at 121 ^O^C. After that, 10 ml of the toxic strain’s spore suspension (10^6^ spores/ml) were added to the corn, and it was incubated for 21 days at 28 to 30 ^O^C to promote fermentation. Following the incubation period, the fungal growth in the corn was then killed in an oven set at 50 ^O^C for three to four days. Then, the corn was crushed and ground into a powder using a grinder. AFB1 was detected in a representative sample of 25 g of powdered corn (A.O.A.C., [1]). The poultry’s basal diet (NRC [47]) (Table 1) was adjusted to include the aflatoxins-contaminated corn at a level that would yield the required dosage of 1 mg of AFB1/kg diet (Zhao et al. [72]). Subsequently, the basal diet was fully homogenized.

**Table 1:** Ingredients and calculated Chemical composition of basal diet

Ingredients (%)	Starter	Grower	Finisher
Yellow Corn (10) %	58.45	63.8	68.2
Soybean meal (44%)	28.3	23.8	20.25
Corn gluten (60%)	8	7	6
Vegetable oil1	1.75	2	2.25
DCP	1.4	1.3	1.3
Limestone	1.4	1.4	1.3
Lysine	0.15	0.2	0.2
DL-Methionine	0.15	0.1	0.1
Choline Chloride	0.1	0.1	0.1
Premix (vitamin)**	0.15	0.15	0.15
Mineral premix	0.1	0.1	0.1
Mycotoxin binder	0.05	0.05	0.05
Total	100	100	100
Chemical composition %			
CP%	23.09	21.05	19.3
ME Kcal/kg diet	2995.8	3050.1	3090.8
Calorie/protein ratio	129.75	144.89	160.14
Ether Extract	4.98	5.65	6.15
Calcium	0.99	0.93	0.89
Total phosphorus	0.69	0.68	0.62
Methionine	0.58	0.47	0.45
Lysine	1.33	1.1	1.04

** The used premix (Multivita Co.) composed of vitamin A 12,000,000 IU, vitamin D3 2,200,000 IU, vitamin E 10,000 mg, vitamin K3 2000 mg, vitamin B1 1000 mg, vitamin B2 5000 mg, vitamin B6 1500 mg, vitamin B12 10 mg, Niacin 30,000 mg, Biotin 50 mg, Folic acid 1000 mg, Pantothenic acid 10,000 mg, Iron 30,000 mg, Manganese 60,000 mg, Copper 4000 mg, Zinc 50,000 mg, Iodine 1000 mg, Cobalt 100 mg, Selenium 100 mg, calcium carbonate (CaCO3) carrier to 3000 g

### Experimental design

This experiment was approved (ARC AHRI 76 23) and conducted at the Animal Health Research Institute, Giza, Egypt. 120 hatched chicks were obtained from local hatchery located in Cairo, Egypt and the authors were granted permission to use the chicks after formally applying to the institute. Chicks were kept under good sanitation and hygienic care in a clean, well-ventilated room (23:1, light: dark). Water and feed were given out at will. Following acclimatization period (3 days), the chicks were divided into 4 group (3 replicates/group and 10 chicks/replicate) by ranking method. The groups were control feed basal diet (Table 1**(**NRC [47]**)**) without additives, Afl group feed diet contaminated with aflatoxin (1 mg of AFB1/kg diet), Afl + Cur group feed diet contaminated with aflatoxin and curcumin as a feed additive (7 g curcumin/kg diet) (Hussein [29]), and Afl + Nano-Cur group feed diet contaminated with aflatoxin and nano curcumin (obtained from Nanotech Company, Egypt.) as a feed additive (400 mg nano curcumin/kg diet) (Ashry et al. [6]). All chicks were vaccinated with Newcastle disease virus and Gumboro vaccines on 12 and 20 days and16 days, respectively. At the start of the trial and once a week thereafter, all the chicks were weighed. To calculate the body weight, weight gain, feed intake, and feed conversion ratio (FCR) along the experimental period (35 days). Throughout the 35-day study period, all groups of chicks were monitored for symptoms, mortality, and postmortem (PM).

### Carcass traits

At the end of the growing period, 3 broilers were randomly selected from each replication (9 broilers/group), fasted for 12 h, and then weighed separately. This process was done to determine the dressing percentage and total edible carcass percentage. Subsequently, the boilers were euthanized with over dose of sodium pentobarbital then slaughtered till they were completely bleeding, then their feathers were plucked. After removing the head and internal organs, the dressed weight was determined by weighing the remaining tissue. Based on the broiler’s final recorded live weight, the dressing % was computed by dividing the carcass weight by the broiler’s final live weight.

### Serum and tissue samples

Three broilers were collected from each replicate (9 broilers/groups) for samples collections. After anesthesia with sodium pentobarbital (50 mg/kg intraperitoneal injection), the blood samples without anticoagulant (5 ml blood/sample/broiler) were collected to separate serum from the wing vein. The levels of serum proteins and aspartate transaminase (AST), alanine transaminase (ALT), and alkaline phosphatase (ALP) activity were determined by using commercial kits (BIODIAGNOSTIC Company, Giza, Egypt).

The tissue samples were liver and muscle for residual estimation by TLC (Schuller and Van Egmond [59]) and liver, spleen, and intestine for histopathology. After necropsy, tissue sections were fixed immediately in 10% buffered formalin and treated with the routine paraffin embedding portion for histopathology evaluation. 3 μm thick sections were cut and stained using H&E (Bancroft and Gamble [8]).

### Statistical analysis

Shapiro-Wilk and Levene tests were employed to demonstrate normality and homoscedasticity before one-way ANOVA was performed to confirm the data’s normal distribution. One-way ANOVA was used to statistically analyze the collected results. Comparison between the groups was performed by Tukey post hoc test. Data were expressed as mean ± SD.

## Results

The prevalence of different mould species isolated from ration samples at Giza and Assiut Governorates was high (Table 2). The higher prevalence occurred at Assiut Governorates.

**Table 2:** Prevalence of different mould species isolated from ration samples from Giza and Assiut governorates

Governorate Isolated mould	Giza Governorate		Assiut Governorate	
	No.	%	No.	%
Penicillium spp.	**27**	**31**	**12**	**12.1**
***Aspergillus spp.***	**39**	**44.8**	**55**	**55.6**
***Fusarium spp***	**11**	**12.6**	**0**	**0**
***Cladosporium spp.***	**3**	**3.5**	**9**	**9.1**
***Scopulariopsis***	**0**	**0**	**6**	**6.1**
***Mucor***	**7**	**8.1**	**10**	**10.1**
***Colletotrichum gloeosporioidides***	**0**	**0**	**4**	**4.0**
***Acremonium strictum***	**0**	**0**	**3**	**3.0**
**Total**	**87**	**100**	**99**	**100**

### Clinical signs and PM lesions

**Fig. 1:**
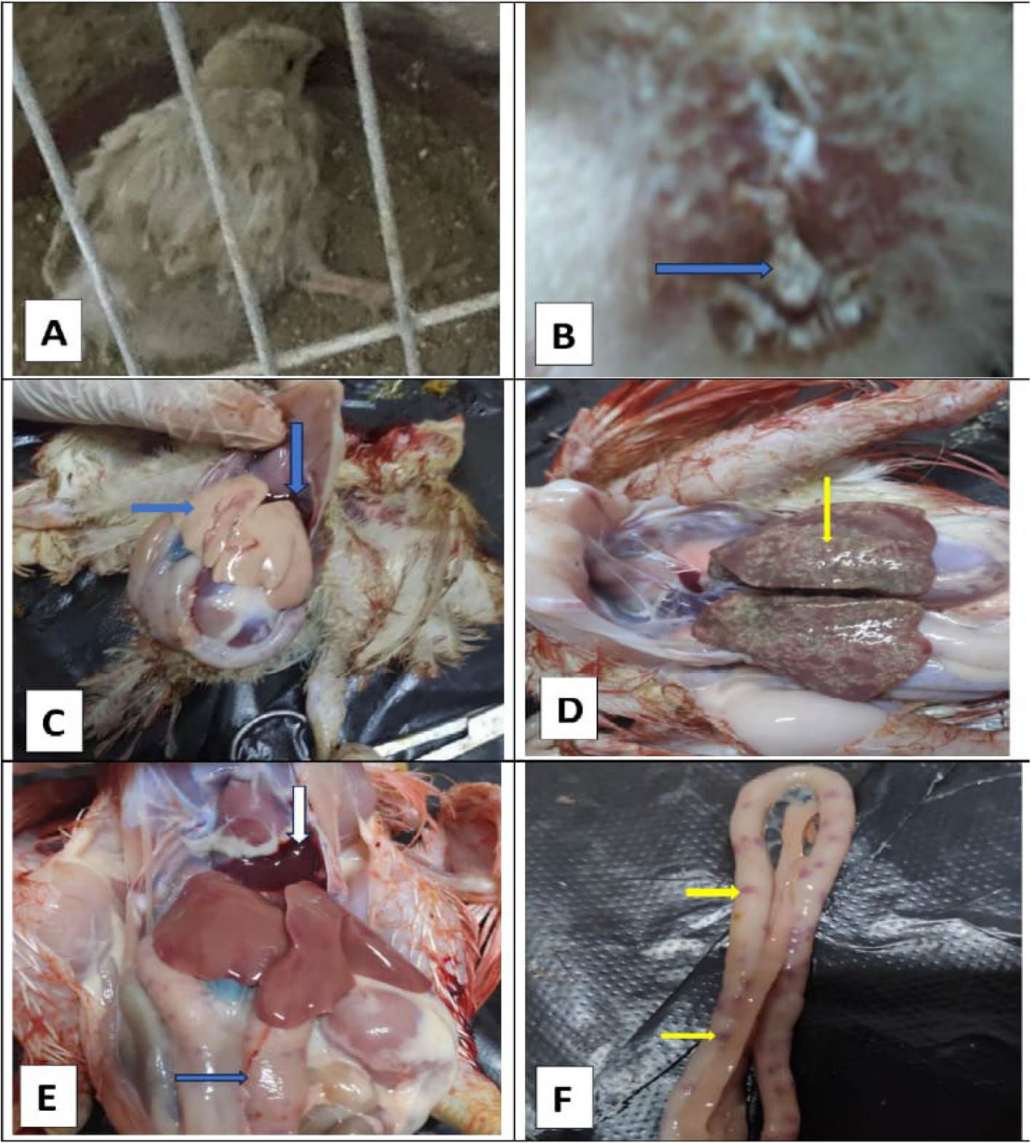
showed clinical and PM lesions of the aflatoxicated broilers. **A** showed leg paralysis. **B** showed whitish diarrhea (arrow). **C** showed enlarged pale-yellow color liver with ascetic fluids (arrows). (D) showed enlargement of the liver size with pen points yellowish discoloration (arrow). **E** showed bleeding around heart with distended intestine (arrows). **F** showed intestine had focal hemorrhage (arrows)

Signs of toxicity with aflatoxin appeared on broilers after 6 days from the starting of the experiment. Signs of toxicity with aflatoxin were retardation in growth, which appeared clearly with the growth parameters (Fig. 1). Leg (Fig. 1A) and wing paralysis was observed with whitish diarrhea (Fig. 1B). Some cases had eye lesions. The mortality rate was higher, about 40%. Some of freshly dead and euthanized chicks showed enlarged pale-yellow color liver with ascetic fluids (Fig. 1C). Moreover, some chicks showed enlargement of the liver size with pen points yellowish discoloration (Fig. 1D). Some cases had bleeding around heart with distended intestine (Fig. 1E). Intestine had focal hemorrhage (Fig. 1F) with enlarged spleen and kidney. Meanwhile, the treated groups (Afl + Cur and Afl + Nano-Cur) not appeared any clinical signs or PM lesions without mortalities (Table 3).

**Table 3:** The mortality rate of broilers fed with aflatoxin contaminated diet, curcumin, and nano curcumin at 35 days

Groups Parameter	Control	Aflatoxin groups		
		Afl	Afl + Cur	Afl + Nano-Cur
Total No.	30	30	30	30
Dead No.	0	12	0	0
Survival %	100	60	100	100
Mortality %	0	40	0	0

### Growth performance

The aflatoxin toxicity affected the growth of the broilers and significantly (*P* ≤ 0.05) decreased the final body weight, weight gain, and feed intake with increased FCR when compared with the control, Afl + Cur, and Afl + Nano-Cur groups. However, the used curcumin and nano curcumin improved the deleterious effects of aflatoxin with superior effect to curcumin (Table 4).

**Table 4:** Growth performance of broiler fed with aflatoxin contaminated diet, curcumin, and nano curcumin at 35 days

Groups Parameters	Control	Aflatoxin groups			*P*-value
		Afl	Afl + Cur	Afl + Nano-Cur	
3-day-old body weight (g/chick)	73.98 ± 0.28	73.80 ± 0.41	73.98 ± 0.34	73.8 ± 0.34	0.964
Final body weight (g/chicks)	1919.6 ± 0.98^a^	1341 ± 1.61^d^	1517.2 ± 1.59 ^b^	1409.32 ± 0.97 ^c^	0.000
Total Weight gain (g/chicks)	1845.62 ± 0.80 ^a^	1267.2 ± 1.45 ^d^	1443.22 ± 1.51 ^b^	1335.52 ± 0.96 ^c^	0.000
Feed intake (g/bird)	3206 ± 2.92 ^a^	2424.4 ± 1.81 ^d^	2643 ± 2.0 ^b^	2465.28 ± 1.68 ^c^	0.000
FCR value	1.74 ± 0.02 ^d^	1.91 ± 0.02 ^a^	1.83 ± 0.02 ^c^	1.85 ± 0.01^b^	0.000

Values are expressed as mean ± standard errors. Means in the same row (a-d) with different letters significantly differ at (*P* ≤ 0.05)

### Carcass traits

The aflatoxin toxicity affected some parameters of broilers carcass traits and significantly (*P* ≤ 0.05) decrease percent of dressing and breast and leg muscles when compared with the control, Afl + Cur, and Afl + Nano-Cur groups. In contrast, liver of Afl group had higher significant (*P* ≤ 0.05) percent when compared with the control, Afl + Cur, and Afl + Nano-Cur groups. On the other hand, the used curcumin significantly (*P* ≤ 0.05) higher percent of dressing and breast and leg muscles when compared with the Afl + Nano-Cur group (Table 5).

**Table 5:** Carcass traits of broiler fed with aflatoxin contaminated diet, curcumin, and nano curcumin at 35 days

Groups Parameters%	Control	Aflatoxin groups			*P*-value
		Afl	Afl + Cur	Afl + Nano-Cur	
Dressing	74.72 ± 0.14 ^a^	51.25 ± 2.50 ^d^	73.50 ± 0.29 ^b^	72.17 ± 0.17 ^c^	0.000
Breast muscle	18.93 ± 0.23^a^	15.4 ± 0.32^d^	18.37 ± 1.52 ^b^	17.6 ± 0.86 ^c^	0.008
Leg muscle	15.9 ± 0.15 ^a^	12.87 ± 0.13 ^d^	15.13 ± 0.13 ^b^	14.3 ± 0.17 ^c^	0.000
Liver	2 ± 0.11 ^d^	2.87 ± 0.09 ^a^	2.36 ± 0.08 ^c^	2.57 ± 0.07 ^b^	0.001

Values are expressed as mean ± standard errors. Means in the same row (a-d) with different letters significantly differ at (*P* ≤ 0.05)

### Biochemistry

**Fig. 2:**
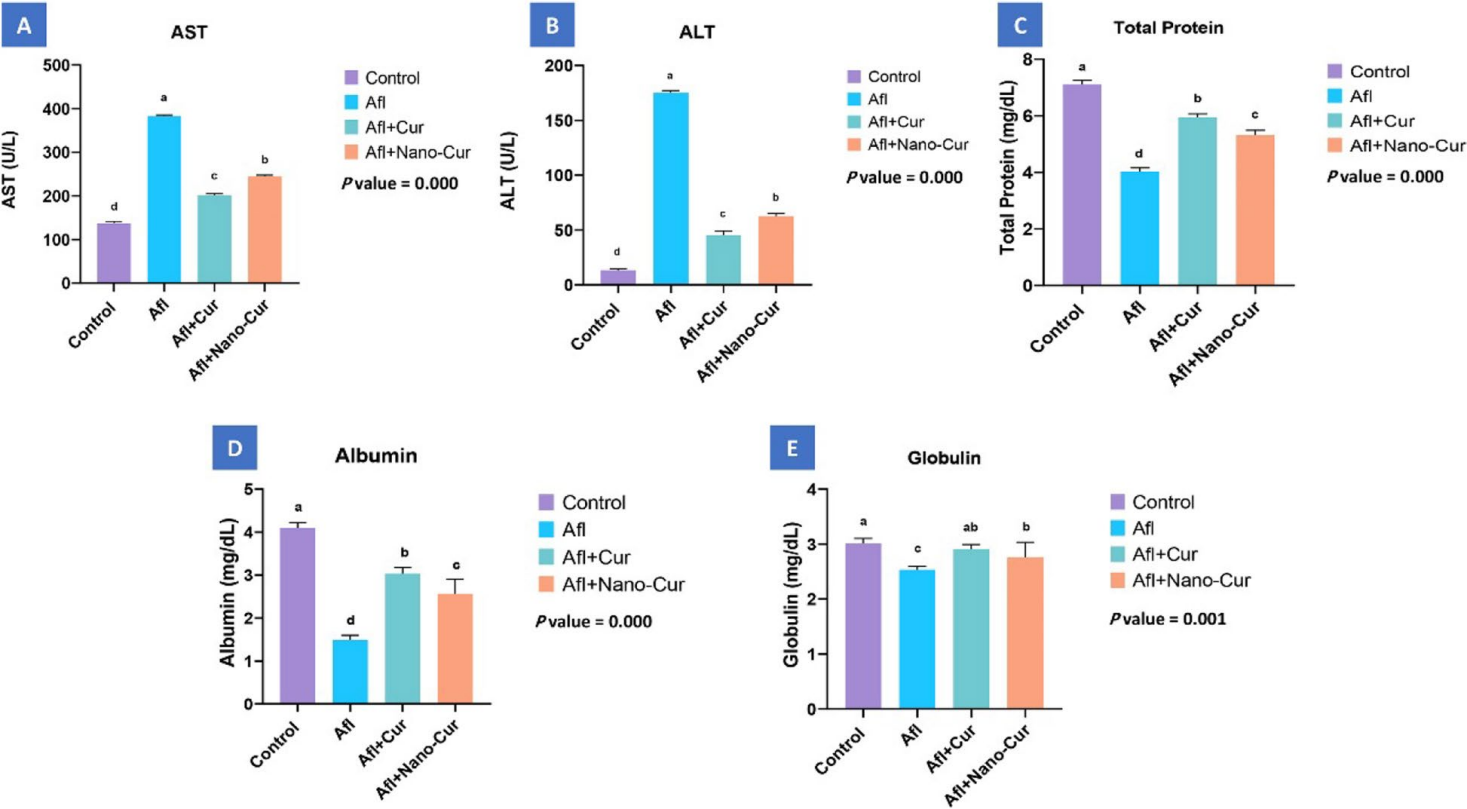
Bar plots of liver parameter levels with Afl, Afl+Cur, and Afl+Nano-Cur exposures (*n* = 9). AST, aspartate aminotransferase; ALT, alanine aminotransferase. Control, aflatoxin (Afl), aflatoxin + curcumin (Afl+Cur), and aflatoxin+nano-curcumin (Afl+Nano-Cur). Significant differences existed across groups with different letters (*P *≤ 0.05)

The aflatoxin toxicity affected the liver function testes of the broilers and significantly (*P* ≤ 0.05) decreased serum proteins with increased ALT and AST when compared with the control, Afl + Cur, and Afl + Nano-Cu groups. However, the used curcumin and nano curcumin improved the deleterious effects of aflatoxin with superior effect to curcumin (Fig. 2).

### Aflatoxin residue

Regarding results of the aflatoxin residues in the liver and muscle tissues, there were higher levels of aflatoxin in the Afl group in both organs. Meanwhile, the residue was not detectable in the control group. However, curcumin reduced these levels significantly (*P* ≤ 0.05) when compared with the nano curcumin (Table 6).

**Table 6:** Aflatoxin B1 residue in the liver and muscle tissues of broiler fed with aflatoxin contaminated diet, curcumin, and nano curcumin at 35 days

Groups Parameters	Control	Aflatoxin groups			*P*-value
		Afl	Afl + Cur	Afl + Nano-Cur	
Residue in the liver tissue (µg/kg)	ND	10.8 ± 0.4^a^	2.3 ± 0.3 ^c^	4.65 ± 0.5 ^b^	0.000
Residue in the muscle tissue (µg/kg)	ND	4.74 ± 0.5 ^a^	1.01 ± 0.2^c^	2.04 ± 0.1 ^b^	0.000

*ND* not detectable AFB1. Values are expressed as mean ± standard errors. Means in the same row (a–c) with different letters significantly differ at (*P* ≤ 0.05)

### Histopathological findings

**Fig. 3:**
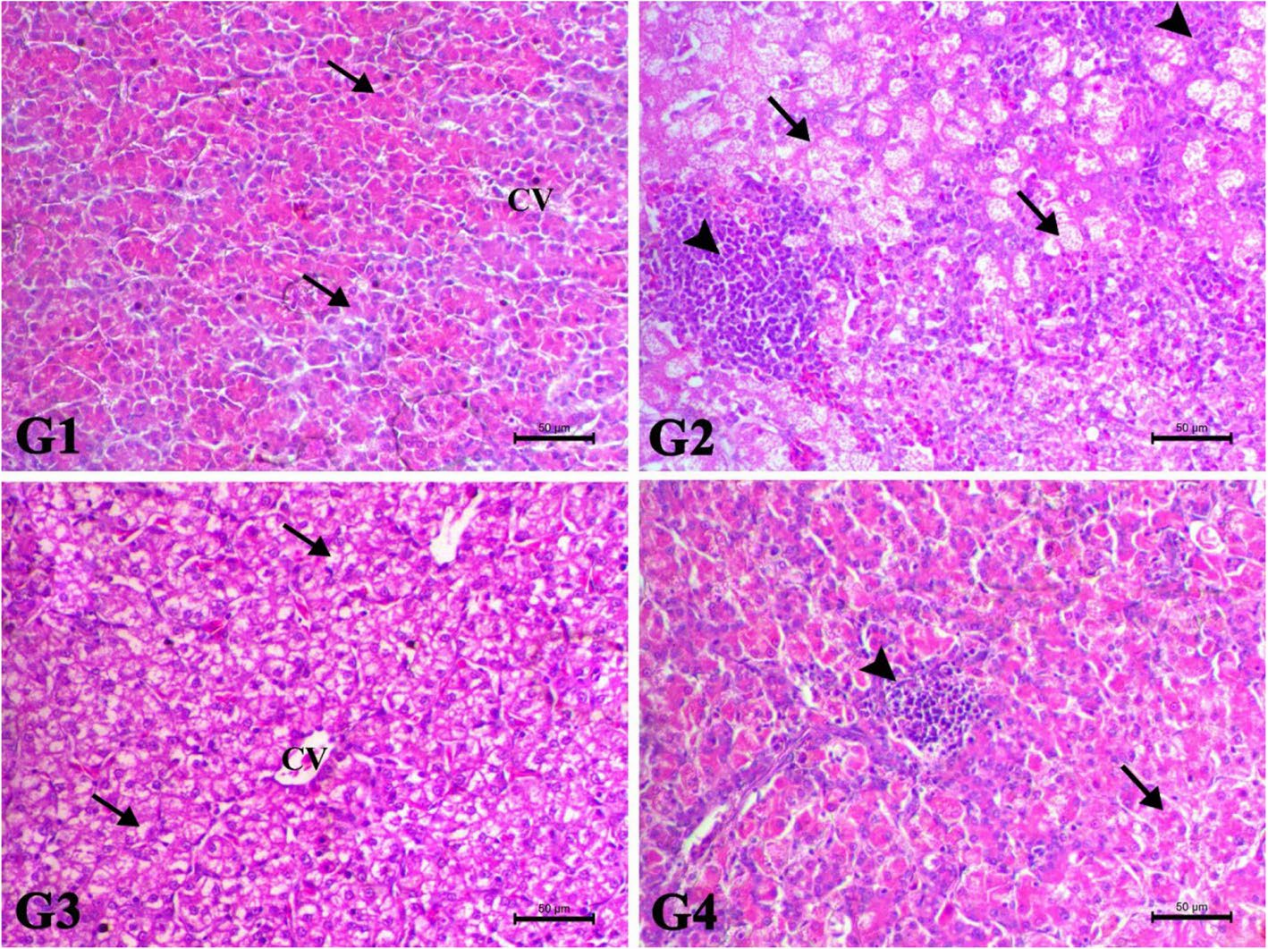
showed the photomicrograph of H&E-stained liver of the different treated groups (X200, Scale bar= 50µ). G1: The liver of control bird showed normal hepatic plates around the central veins. The hepatocytes showed normal esinophilic cytoplasm with normal vesicular nucleus (arrows). G2: The Afl group showed marked clear hepatic atypia associated with severe hepatic ballooning. Some of hepatic cells showed basophilic regenerative changes. There was also marked periportal and multifocal mononuclear cells infiltration mostly lymphocytes, macrophages and with few heterophils (arrows). G3: The Afl+Cur group revealed marked decrease cytoplasmic atypia, degenerative and inflammatory changes. Most of hepatic cells showed mild vacuolar degenerative changes. G4: The Afl+Nano-Cur showed decrease of degenerative changes with mild to moderate degree of periportal mononuclear cells infiltration mostly lymphocytes

The liver of control bird showed normal hepatic plates around the central veins. The hepatocytes showed normal esinophilic cytoplasm with normal vesicular nucleus. The Afl group showed marked clear hepatic atypia associated with severe hepatic ballooning. Some of hepatic cells showed basophilic regenerative changes. There was also marked periportal and multifocal mononuclear cells infiltration mostly lymphocytes, macrophages and with few heterophils. The Afl + Cur group revealed marked decrease cytoplasmic atypia, degenerative and inflammatory changes. Most of hepatic cells showed mild vacuolar degenerative changes. The Afl + Nano-Cur showed decrease of degenerative changes with mild to moderate degree of periportal mononuclear cells infiltration mostly lymphocytes (Fig. 3).

**Fig. 4:**
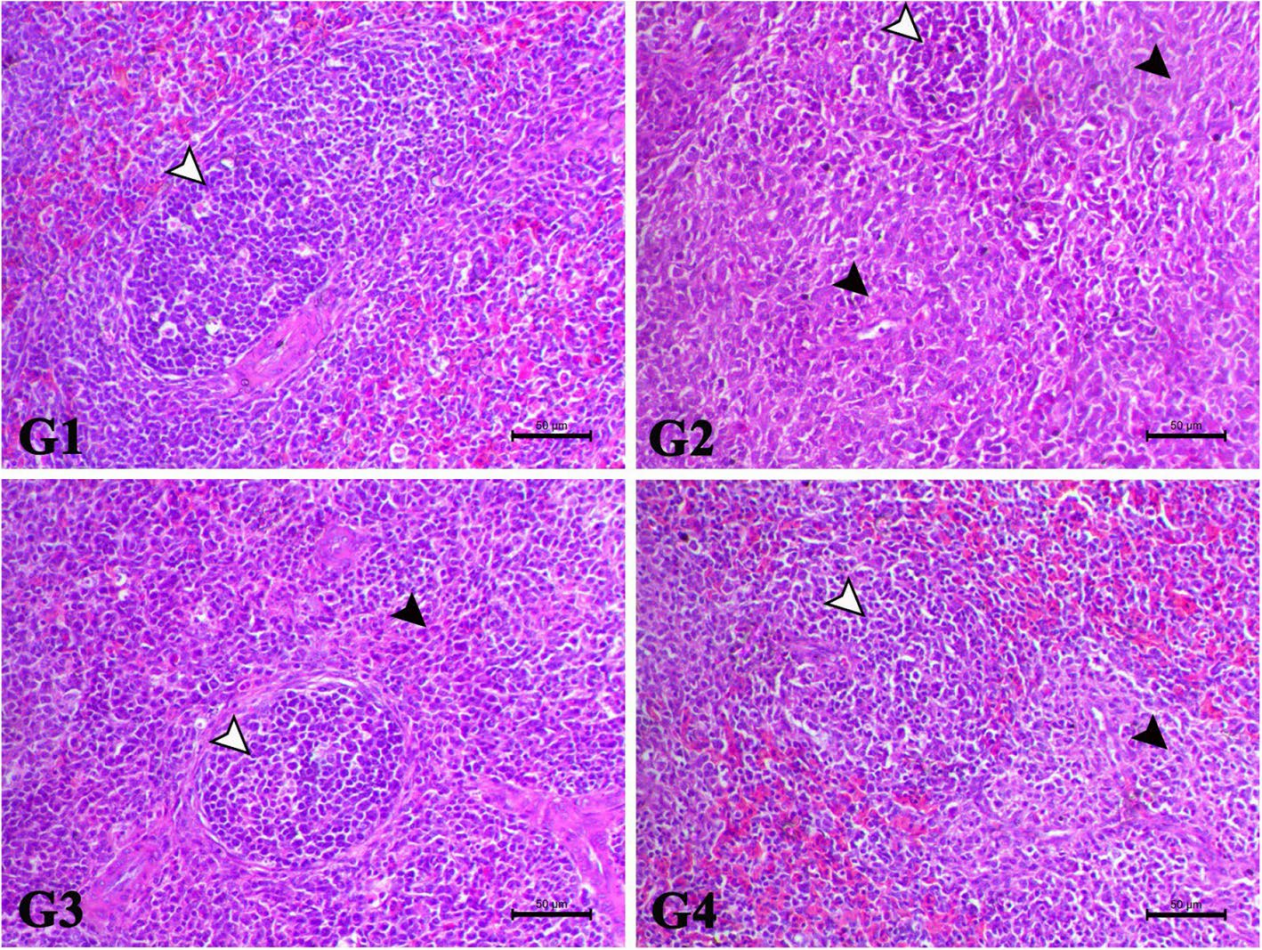
showed the photomicrograph of H&E-stained spleen of the different treated groups (X200, Scale bar= 50µ). G1: The control bird showed normal white and red pulps with normal lymphoid follicles within the white pulp portion. G2: The spleen of Afl group showed marked lymphoid depletion with marked diffuse histocytes cells infiltration replacing whole lymphoid parenchyma. G3: The spleen of Afl+Cur group demonstrated marked increase of the lymphoid cells with normal lymphoid follicles. G4: The spleen of Afl+Nano-Cur showed increase the lymphocytes within the lymphoid cells with multifocal histocytic cells infiltration within the splenoic parenchyma

The spleen of the control bird showed normal white and red pulps with normal lymphoid follicles within the white pulp portion. The spleen of Afl group showed marked lymphoid depletion with marked diffuse histocytes cells infiltration replacing whole lymphoid parenchyma. The spleen of Afl + Cur group demonstrated marked increase of the lymphoid cells with normal lymphoid follicles. The spleen of Afl + Nano-Cur showed increase the lymphocytes within the lymphoid cells with multifocal histocytic cells infiltration within the splenoic parenchyma (Fig. 4).

**Fig. 5:**
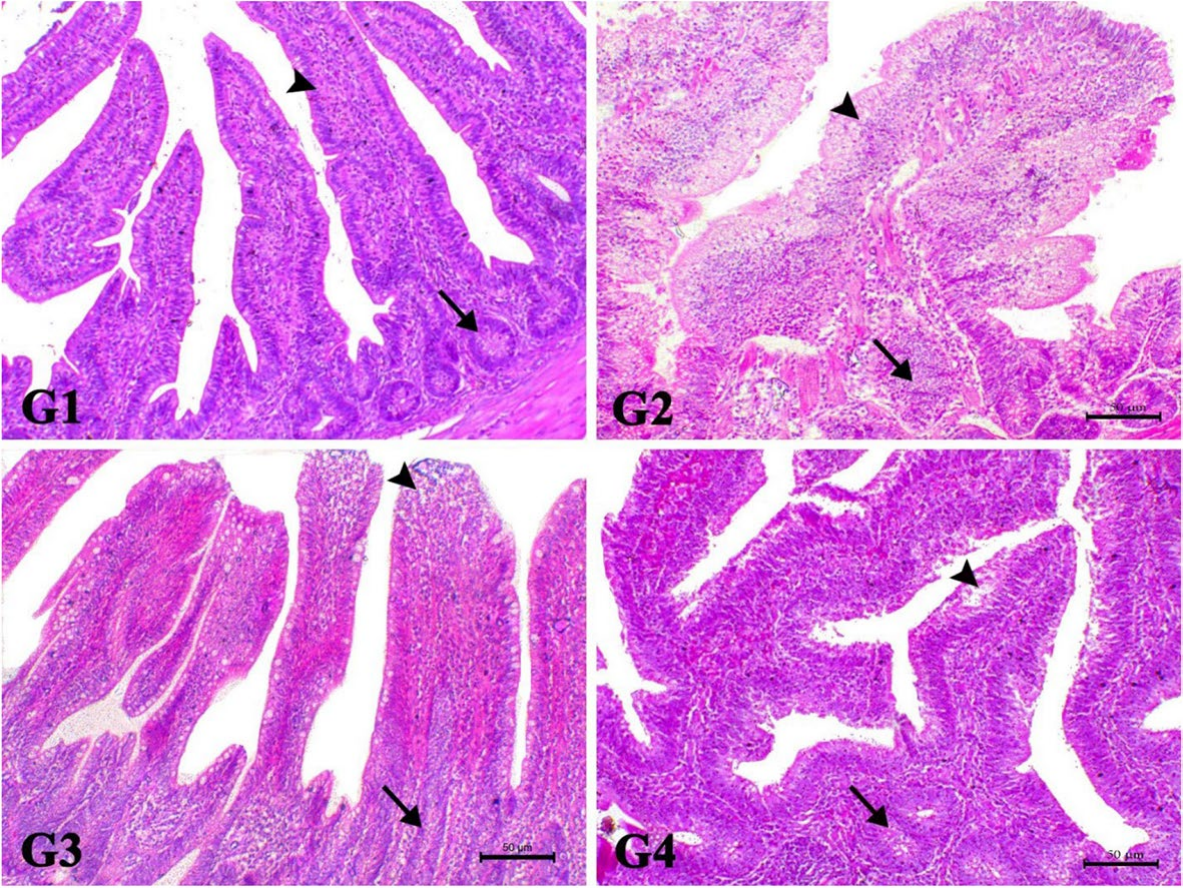
showed the photomicrograph of H&E-stained intestine of the different treated groups (X200, Scale bar= 100µ). G1: The intestine of the control bird showed normal intestinal villi. G2: The intestine of Afl group showed marked enteritis features associated with blunting of the villi and marked hyperplasia of goblets cells. G3: The intestine of Afl+Cur group demonstrated marked decrease the enteritis features and marked increase of villi length. G4: The intestine of Afl+Nano-Cur showed an increase the length of the intestinal villi

The intestine of the control bird showed normal intestinal villi. While the intestine of Afl group showed marked enteritis features associated with blunting of the villi and marked hyperplasia of goblets cells. The intestine of Afl + Cur group demonstrated marked decrease the enteritis features and marked increase of villi length. The intestine of Afl + Nano-Cur showed an increase the length of the intestinal villi (Fig. 5).

## Discussion

Prevalence of different mould species in poultry feed samples examined for this investigation indicated a significant occurrence of mould species, which is consistent with findings from Torky et al. ([66]), Hassan et al. ([24]), and Mahmoud and Mohamed ([37]**)**, who reported high mould incidence in poultry diet in the Governorates of Assiut, Ismailia, and Dakhalia, respectively. Moreover, Ghada and Emtenan ([18]**)** found that all collected samples at Beni-Suef Governorate, poultry diet or liver, have aflatoxin and ochratoxin. The higher incidence of the mould species in Assiut Governorate may be attributed to high humidity and temperature. As per Guynot et al. ([23]) and Giorni et al. ([20]), the primary determinants of growth and mycotoxin production are temperature of the surrounding environment, substrate water activity, relative humidity, gas composition, substrate composition, inoculum concentrations, microbial interactions, and mechanical or insect damage. Specifically, whether contamination rises and mycotoxin production occurs depends on how some or all of these elements interact. Because fungi have the ability to germinate, grow, and actively compete for the allocation of available resources, interactions between temperature and accessible water are crucial (Samapundo et al. [58] and Marín et al. [40]).

The clinical signs of aflatoxin toxicity in this investigation are constant with the clinical signs of Ashry et al. ([6]) and Salem et al. ([57]), who noticed eye lesions and paralysis of leg and wing with retarded growth. In constant, the mortality rate in this study very high that could be attributed to the high dose of aflatoxin. However, the acute toxicity was pronounced in the mortality rate and gross lesions. Pale, friable, and enlarged livers with severe mucosal congestion in the intestines were the primary abnormalities found in necropsied broilers, along with an excess of catarrhal exudate in the intestinal lumen (Mariappan et al. [39]). Moreover, bleeding was noticed by Baily et al. ([7]) and Makinia ([38]**)** in poultry. These signs and PM lesions ameliorated by curcumin inclusion in the poultry diets. Similar findings were observed by Ashry et al. ([6]) and Sadiek et al. ([55]) with nano curcumin and curcumin, respectively. They found that aflatoxin’s detrimental effects on broilers performance, target organ histopathology, and serum biochemical markers were lessened by nano curcumin and curcumin. Raja et al. ([52]) reported that the addition of curcumin to broilers diet may help reduce poultry aflatoxicity to some extent. It has been established that curcumin has the ability to lessen the negative effects of animal stress caused by toxicity, inflammation, illnesses, heat effect, and other factors (Tuong et al. [67]).

The reduction of performance metrics is one of the most economically significant impacts of AFB1 in broilers (Resanović et al. [54]). The results of the clinical signs were confirmed by results of growth and histopathology. Aflatoxin retarded growth of the broilers in the present study as indicated by lowered body weight, weight gain, and feed intake with higher FCR. Aflatoxin dietary contamination affect the feed intake of broilers as reported by Nazarizadeh et al. ([46]), Alam et al. ([3]), and Alharthi et al. ([4]). In contrast, Denli et al. ([11]), Chen et al. ([10]), and Rashidi et al. ([53]) reported that AF contaminated ration did not affect significantly feed intake of broilers. However, the decreased in quantity of consumed feed was reflected in a decrease in weight gain of broilers in this study. These results are confirmed by the findings of Bhatti et al. ([9]), Nazarizadeh and Pourreza ([45]**)**, and Alam et al. ([3]) and Khaleghipour et al. ([31]) in broilers and quail, respectively. Moreover, The FCR is a ratio or rate used in poultry production to measure how well the bodies of the birds convert animal feed into the intended product (Fawaz et al. [15]). The present study showed significant lower intended product of the aflatoxicated group that indicated by increase FCR. Ashry et al. ([6]) and Salem et al. ([57]) and Fadl et al. ([14]) found the same results in the broilers and Nile tilapia, respectively. Moreover, Alharthi et al. ([4]), Solis-Cruz et al. ([62]), and Liu et al. ([36]) reported that FCR increased in broilers fed aflatoxin contaminated ration. In contrast, Tedesco et al. ([64]) and Nazarizadeh et al. ([46]) found that aflatoxin not affected FCR of broilers. Khaleghipour et al. ([31]) reported that FCR of quails not affected by dietary aflatoxin. This difference may be returned to species and sex of birds used and period of aflatoxin exposure. However, the retarded growth partially ameliorated by curcumin and nano curcumin inclusion. These were confirmed by result of Gowda et al. ([22]), who noticed that *Curcuma longa* added to the AFB1 diet partially increased the broiler’s feed efficiency and weight gain. It has already been documented that *Curcuma longa* provides partial protection against AFB1 (Gowda et al. [21]). Moreover, Ashry et al. ([6]) reported that nanocurcumin enhanced growth of aflatoxicated broilers. Anyway, the better effect was to curcumin group about nano group. These effects can be attributed to the effect of heat and light during nano curcumin preparation where curcumin is extremely susceptible to heat, light, and an alkaline pH (Gowda et al. [22]).

The negative effects of aflatoxin on the growth reflected on the carcass traits, especially breast and leg muscle with enlarged liver weight. Comparable results were obtained by Ashry et al. ([6]). Feed tainted with aflatoxin interferes with bone metabolism, reducing bone strength, diameter, dressed weight, and breast production, among other effects (Morrison et al. [42]). Alam et al. ([3]) reported that aflatoxin reduced dressing of broilers. Furthermore, Zaker-Esteghamati et al. ([70]) documented aflatoxin’s detrimental effects on gut microbiota, carcass and meat qualities, and growth performance. Aflatoxin negatively affects the relative weight of organs (Jahanian et al. [30]), particularly the liver tissue (Dhanaoal et al. [12]; Saleemi et al. [56]). In contrast, Nazarizadeh et al. ([46]) found that broiler’s liver weight decreased with aflatoxin. On the other hand, Tessari et al. ([65]) reported that aflatoxin not affected the liver weight of broilers. Anyway, dietary curcumin and nano curcumin decreased relative weight of the liver and agreement with the results of Ashry et al. ([6]). While, Yarru et al. ([69]) reported that relative weight of liver insignificantly decreased with curcumin inclusion in the diet of aflatoxicated poultry.

Since the liver is the primary organ targeted by AFB1, hepatic damage would result from AFB1 poisoning (Zhang et al. [71]). The effect of aflatoxin on the liver function testes was confirmed the result of the relative liver weight in the aflatoxicated group. Moreover, the toxic effects of AFB1 on hepatic tissue are shown by decreased levels of total protein, albumin, and globulin and elevated levels of ALT and AST. Rashidi et al. ([53]) and Denli et al. ([11]) and Khaleghipour et al. ([31]) reported that serum liver enzymes increased significantly in the aflatoxicated group of broilers and quails, respectively. In contrast, Nazarizadeh and Pourreza ([45]**)** found that dietary aflatoxin not affected liver enzymes of broilers significantly. It is well known that AFs inhibit protein synthesis, which could result in lower blood protein levels (Allah Ditta et al. [5]). Dönmez and Keskin ([13]**)** reported that AF decreased serum total protein. AFs reduce total blood proteins by reducing α, β, and γ globulins (Allah Ditta et al. [5]). Comparable results were obtained by Ashry et al. ([6]) and Salem et al. ([57]). Anyway, dietary curcumin and nano curcumin improved these altered effects on the serum proteins and liver enzymes. The altered activity of ALT by dietary aflatoxin was returned to normal when curcumin was added to the diet of chicken **(**Nayak and Sashidhar [44]**)**. Comparable results were obtained by Ashry et al. ([6]) with nano curcumin in broilers. These results could be returned to the antioxidant effect of curcumin and nano curcumin. Curcumin has antioxidant properties that protect against oxidative stress brought on by free radicals (Okada et al. [48]). Curcumin has the ability to lower levels of inflammatory cytokines and indicators of oxidative stress (Li et al. [34]). Moreover, curcumin is known to shield the liver from AFB1 by preventing AFB1 from being biotransformed into aflatoxicol in the liver (Lee et al. [33]; Soni et al. [63]). Curcumin works well to lessen the toxicity caused by AFB1 (Pauletto et al. [49]; Muhammad et al. [43])).

The present study showed that aflatoxin residue in the liver and muscle tissue of broilers, serum parameters, growth performance, and carcass features were all negatively impacted by aflatoxin inclusion. These results were confirmed by Ashry et al. ([6]) and Salem et al. ([57]), who reported aflatoxin residue in the liver and muscle and liver tissues of broilers, respectively. This is consistent with other researches Kumar and Balachandran ([32]**)**, Herzallah et al. ([26]) and Yang et al. ([68]) in broilers. In contrast, Hussain et al. ([28]) not detected aflatoxin residue in broilers. This difference could be returned to the aflatoxin dose that used in the study of Hussain et al. (50 ppb AFB1). The aflatoxin residue decreased with curcumin and nano curcumin inclusion. Comparable results were obtained by Ashry et al. ([6]) and Abd-Elwahab et al. ([2]), who noticed that decreased aflatoxin residue with nano curcumin and curcumin inclusion in the diet of broilers and quails, respectively.

All the above-mentioned parameters were confirmed by the results of histopathology. Where serious lesions in the intestinal, hepatic, and splenic tissues of chickens were caused by dietary AFB1. Similar results were obtained by Salem et al. ([57]) and Ashry et al. ([6]) in broilers. On the other hand, curcumin administration decreased the histological alterations brought on by AFB1 (Li et al. [34]). This may be attributed to the large dose of aflatoxin used in this study. This is consistent with other research that demonstrated curcumin can both prevent and treat liver damage caused by AFB1 in chickens (Nayak and Sashidhar [44]; Zhang et al. [71]). Moreover, Ashry et al. ([6]) reported that nano curcumin ameliorated the deleterious effects of aflatoxin on organs pathology.

### Public health and socio-economic impact

This research had public health, social, and economic importance. Aflatoxin has a negative impact on the health of both humans and animals. Limaye et al. ([35]) reported that food contamination by various mycotoxin components results in serious health issues and financial losses for both people and animals. In poultry production, a lower growth rate and frequent broilers deaths are the economically detrimental results of aflatoxicosis in chickens (Salem et al. [57]). In this study, broilers meat retained aflatoxin, this issue will affect those who eat these meats. For human, in addition to contaminated fruits and crops, aflatoxin residue in the animal meat can also introduce them into the food chain (Fawaz et al. [15]). According to the previous discussion, curcumin enhanced growth performance and reduced aflatoxin residue, thus it may be possible to overcome this financial failure. As a result, it may have a favorable impact on economics and human health.

## Conclusion

The current study offers an experimental scientific foundation for the use of curcumin as a medicinal medication or supplement in animal husbandry practices. Where the findings of this experiment showed the ameliorated effects of curcumin to deleterious effects of aflatoxin, although large doses of aflatoxin used. These pronounced in the growth parameters, carcass traits, aflatoxin residue, and histopathology of different tissues.

## Data Availability

The datasets used and/or analyzed during the current study are available from corresponding author on reasonable request.
